# The Diagnosis and Treatment of Chronic Nasal Catarrh

**Published:** 1885-02

**Authors:** 


**The Diagnosis and Treatment of Chronic Nasal Catarrh.** Three Clinical Lectures, delivered at the College of Physicians and Surgeons, New York. By GEORGE MOREWOOD LEFFERTS, A. M., M. D., Professor of Laryngoscopy and Diseases of the Throat in the College of Physicians and Surgeons. Reprinted from the *Medical News* of Philadelphia. St. Louis: Lambert & Co. 1884.

These lectures treat of the methods of examination of the nasal passages and pharynx, diagnosis, including the instruments for diagnosis, the treatment and appliances for treatment, etc. The work is a practical one, written by one of the most able of the specialists, and is just such a compendium as the physician needs who aims to acquire a practical knowledge of this class of diseases. We recommend it to our readers.

